# Adjunctive Therapies in Long-Bone Distraction Osteogenesis: Clinical Evidence for Biophysical and Biologic Treatment Strategies

**DOI:** 10.3390/jcm15124417

**Published:** 2026-06-07

**Authors:** Waleed Albishi, Omar A. Aldosari, Abdulmalik Alduraibi, Abdulaziz S. AlNahari, Abdullah I. Alturki, Othman O. Aldraihem, Fahad Alshayhan

**Affiliations:** 1 Department of Orthopedic Surgery, College of Medicine, King Saud University, Riyadh 12372, Saudi Arabia; 2 College of Medicine, King Saud University, Riyadh 11461, Saudi Arabia

**Keywords:** distraction osteogenesis, low-intensity pulsed ultrasound, platelet-rich plasma, bone marrow aspirate concentrate, bone regeneration, healing index

## Abstract

**Objectives:** Distraction osteogenesis (DO) is an established technique for bone regeneration but is associated with prolonged consolidation time and extended external fixation. Biophysical and biologic adjuncts have been proposed to accelerate regenerative maturation. This systematic review aimed to comparatively evaluate the available clinical evidence regarding low-intensity pulsed ultrasound (LIPUS) and biologic augmentation strategies in distraction osteogenesis. **Methods:** A systematic review was conducted in accordance with PRISMA 2020 guidelines and prospectively registered in PROSPERO (CRD420251125456). MEDLINE, Embase, Scopus, and Google Scholar were searched from inception to October 2025. Randomized controlled trials and cohort studies evaluating LIPUS, platelet-rich plasma (PRP), bone marrow aspirate concentrate (BMAC), culture-expanded mesenchymal stem cells, or hyperbaric oxygen therapy in distraction osteogenesis were included. Risk of bias was assessed using RoB 2 for randomized trials and structured domain-based criteria for observational studies. Due to substantial clinical and methodological heterogeneity, findings were synthesized narratively. **Results:** Nine studies involving 304 participants met the inclusion criteria, including randomized controlled trials and cohort studies across multiple anatomical sites and fixation techniques. Randomized trials evaluating LIPUS demonstrated inconsistent reductions in healing index and consolidation time, with no consistent effect on complication rates. Biologic adjuncts such as PRP, BMAC, and cell-based therapies showed signals of improved consolidation parameters in selected studies; however, evidence was limited by small sample sizes and methodological heterogeneity. Hyperbaric oxygen therapy lacked sufficient high-quality evidence to support routine use. Overall, the certainty of evidence was constrained by variability in study design, outcome definitions, and risk of bias. **Conclusions:** Although both biophysical and biologic adjuncts demonstrate compelling biological rationale, current clinical evidence in distraction osteogenesis remains heterogeneous and inconclusive. Biologic strategies may offer theoretical advantages through direct cellular and growth factor supplementation, whereas LIPUS provides non-invasive mechanotransductive stimulation; however, neither approach can currently be recommended for routine clinical use. High-quality, adequately powered trials with standardized outcome reporting are required to define their true clinical role. **Level of Evidence:** Level III (Systematic review of Level I–III studies).

## 1. Introduction

Distraction osteogenesis (DO) is a biologically driven bone regeneration technique in which osteotomy followed by gradual mechanical distraction induces the formation of new, vascularized bone between two progressively separated segments [1]. The biological foundation of DO lies in mechanotransduction—the conversion of mechanical strain into intracellular biochemical signaling [2,3]. Clinically, DO progresses through latency, distraction, and consolidation phases, culminating in regenerative maturation and remodeling [1].

Despite its robust biological basis, distraction osteogenesis remains clinically demanding due to prolonged treatment duration and extended reliance on external fixation. Treatment often requires prolonged external fixation, frequently extending over several months [4]. Prolonged fixation is associated with considerable morbidity. Numerous complications may occur during limb lengthening, particularly with prolonged fixation [4]. Pin tract infection remains the most frequent adverse event, with reported incidence ranging from 10% to over 95%, and is strongly correlated with duration of fixator application [5]. These challenges underscore the importance of strategies aimed at accelerating regenerative maturation and reducing external fixation duration.

Low-intensity pulsed ultrasound (LIPUS) has emerged as a non-invasive biophysical modality intended to enhance bone regeneration through mechanotransduction [6,7]. It represents one component of a broader spectrum of biophysical stimulation strategies under investigation for bone regeneration, which also includes emerging modalities such as wireless bioelectric stimulation using magnetoelectric materials [8]. Given that distraction osteogenesis relies on strain-induced cellular activation and coordinated angiogenic–osteogenic coupling, LIPUS represents a biologically plausible adjunct during distraction and consolidation phases. Early randomized trials in fracture healing suggested reductions in time to union with LIPUS [9]. However, subsequent large-scale studies and systematic reviews have questioned its clinical efficacy, reporting inconsistent effects on radiographic healing and functional recovery, thereby generating ongoing controversy regarding its true therapeutic value [10,11,12].

In contrast to biophysical stimulation, biologic adjuncts aim to directly augment the cellular and molecular environment of the distraction gap. Platelet-rich plasma (PRP) has been explored as a biologic adjunct to enhance bone regeneration [10]. Cell-based therapies such as bone marrow aspirate concentrate have also been investigated [13,14,15,16]. Hyperbaric oxygen therapy has similarly been proposed to support regenerative maturation [17,18,19,20]. Targeted delivery of osteogenic growth factors such as insulin-like growth factor-I through adaptive hydrogel systems has also emerged as a promising strategy for enhancing pediatric bone regeneration and growth plate remodeling [21]. Collectively, these biologic strategies represent mechanistically distinct approaches to enhancing bone regeneration by amplifying growth factor signaling, supplying progenitor cells, or modulating the metabolic microenvironment. These approaches complement broader advances in bone tissue engineering, including scalable scaffold-based strategies and biologically active matrices that aim to recapitulate the native osteogenic microenvironment [22].

Despite compelling biological rationale, clinical evidence supporting adjunctive therapies in distraction osteogenesis remains heterogeneous and inconclusive. Studies vary widely in anatomical site, fixation method, distraction protocol, adjunct type, and outcome reporting, limiting direct comparison and precluding consistent conclusions regarding their relative efficacy. While LIPUS has been extensively investigated in fracture healing with mixed results, and biologic strategies such as PRP, BMAC, cell-based therapies, and HBO demonstrate promising regenerative potential, no structured synthesis has comprehensively compared biophysical and biologic adjuncts within the context of distraction osteogenesis.

Therefore, the aim of this systematic review was to evaluate and compare the available clinical evidence regarding low-intensity pulsed ultrasound and biologic adjuncts in distraction osteogenesis, with particular emphasis on healing indices, consolidation time, complication rates, and overall treatment efficiency.

## 2. Methods

### 2.1. Protocol and Registration

This systematic review was conducted in accordance with the Preferred Reporting Items for Systematic Reviews and Meta-Analyses (PRISMA) 2020 guidelines. The protocol was prospectively registered with the International Prospective Register of Systematic Reviews (PROSPERO; registration number CRD420251125456). Eligibility criteria included patients undergoing distraction osteogenesis who underwent biophysical or biologic adjuncts compared to standard DO; outcomes include healing index, consolidation time, complications, and functional outcomes; and detailed eligibility criteria are explained below.

### 2.2. Population

We included human participants of any age undergoing distraction osteogenesis (DO) for any clinical indication, including limb lengthening, deformity correction, treatment of bone defects, alveolar or craniofacial reconstruction, and management of non-union. Only studies in which distraction osteogenesis constituted the primary bone regeneration technique were considered eligible. Animal and in vitro studies were excluded. Studies evaluating bone regeneration procedures not based on gradual distraction osteogenesis (such as acute fracture healing or spinal fusion without distraction) were also excluded. Additionally, studies including mixed populations were excluded if data specific to distraction osteogenesis could not be extracted separately. Patients with systemic conditions known to severely impair bone healing, such as uncontrolled diabetes, active malignancy, or severe osteoporosis, were excluded unless reported as a clearly extractable subgroup. This approach allowed inclusion of different anatomical sites while maintaining focus on biologically mediated bone regeneration through distraction.

### 2.3. Interventions

Eligible interventions included biologic or biophysical adjuncts applied during the distraction and/or consolidation phases of distraction osteogenesis. Specifically, studies investigating low-intensity pulsed ultrasound (LIPUS), platelet-rich plasma (PRP), bone marrow aspirate concentrate (BMAC), culture-expanded bone marrow–derived mesenchymal stem cells, and hyperbaric oxygen therapy (HBO) were included. Studies utilizing ultrasound modalities other than LIPUS (such as continuous or high-intensity ultrasound) were excluded. Similarly, platelet derivatives other than PRP—such as platelet-rich fibrin or platelet lysate—were not considered eligible in order to maintain comparability across interventions.

### 2.4. Comparators

The comparator was distraction osteogenesis performed without adjunctive biologic or biophysical enhancement (standard DO protocol).

### 2.5. Study Design

Randomized controlled trials and cohort studies (prospective or retrospective) conducted in clinical or hospital settings were eligible. No restrictions were placed on geographic location or healthcare setting.

### 2.6. Information Sources and Search Strategy

A comprehensive literature search was performed in MEDLINE, Embase, Scopus, and Google Scholar from database inception until the sixth of October 2025. Only studies published in English were considered. The gray literature and unpublished studies were not actively sought. Distraction osteogenesis research requires detailed reporting of surgical techniques, distraction parameters, and radiographic consolidation outcomes to allow meaningful comparison and risk-of-bias assessment. Non-peer-reviewed sources frequently lack sufficient methodological transparency and standardized outcome reporting, limiting data reliability and extractability. Furthermore, distraction osteogenesis studies are typically small investigator-initiated clinical series rather than industry-sponsored trials, making the likelihood of large unpublished datasets low. Given the planned narrative synthesis and absence of quantitative pooling, restriction to the peer-reviewed literature was considered appropriate to maintain methodological rigor and reproducibility.

To ensure the retrieval of all relevant articles in the literature, we searched for each modality separately. The search strategies were: (Low-intensity pulsed ultrasound OR LIPUS) AND (distraction osteogenesis) AND (Randomized clinical trials OR cohort study); (Platelet-rich plasma OR PRP) AND (distraction osteogenesis) AND (Randomized clinical trials OR cohort study); (Bone marrow aspirate concentrate OR BMAC OR mesenchymal stem cells) AND (distraction osteogenesis) AND (Randomized clinical trials OR cohort study); and (Hyperbaric oxygen OR HBO) AND (distraction osteogenesis) AND (Randomized clinical trials OR cohort study).

### 2.7. Study Screening and Selection Process

Two reviewers independently screened titles and abstracts for eligibility. Potentially relevant articles underwent full-text review. Disagreements were resolved through discussion and consensus. The study selection process is illustrated in the PRISMA flow diagram.

### 2.8. Data Extraction

Data extraction was performed independently by two reviewers using a standardized data collection form. Extracted variables included study design and setting, participant demographics, indication for distraction osteogenesis, anatomical site, fixation method, adjunct protocol (including type, dose, and timing), distraction parameters, healing indices, consolidation time, reported complications, radiographic outcomes, and functional or patient-reported outcome measures. Any discrepancies between reviewers were resolved through discussion and consensus.

### 2.9. Outcomes

#### 2.9.1. Primary Outcome

The primary outcome was reduction in treatment duration, assessed using the healing index (HI), defined as the time required for bone consolidation relative to the size of the distraction gap and expressed as days per centimeter, when applicable.

#### 2.9.2. Secondary Outcomes

Secondary outcomes included complication rates (such as pin-tract infection, delayed consolidation, pseudoarthrosis, and refracture), radiological indicators of bone regeneration, bone density changes assessed through radiographic methods, functional outcomes, and patient-reported outcome measures.

### 2.10. Risk of Bias Assessment Methodology

Risk of bias was assessed independently by two reviewers. Randomized controlled trials were evaluated using the Cochrane Risk of Bias 2 (RoB 2) tool. Non-randomized studies were assessed using the Risk Of Bias In Non-randomized Studies of Interventions (ROBINS-I) tool. Each study was judged as having low risk, some concerns, or high risk of bias for randomized trials, and low, moderate, serious, or critical risk of bias for non-randomized studies. Any disagreements between reviewers were resolved through discussion or consultation with a third reviewer.

### 2.11. Data Synthesis

A quantitative meta-analysis was planned when studies were sufficiently homogeneous in terms of population, anatomical site, intervention type, comparator, and outcome definitions, using a random-effects model.

However, outcome reporting demonstrated marked inconsistency. Healing indices were variably defined as consolidation time per centimeter, external fixation index, or regenerative maturation index, and were reported using heterogeneous units (days/cm, months/cm, cortical-specific indices, or non-convertible measures). Several studies lacked measures of variance or reported outcomes qualitatively, precluding calculation of pooled effect estimates. Given the substantial clinical heterogeneity in patient populations, anatomical sites, fixation strategies, distraction protocols, and outcome definitions, together with the limited number of methodologically comparable studies per intervention category, the prespecified conditions for quantitative pooling were not met. Because pooling under such conditions risks generating misleading summary estimates, quantitative synthesis was not performed.

Accordingly, findings are presented as a structured narrative synthesis organized by adjunct type and outcome category, in accordance with PRISMA 2020 recommendations (Supplementary Materials). 

## 3. Results

### 3.1. Study Selection

**Figure 1:**
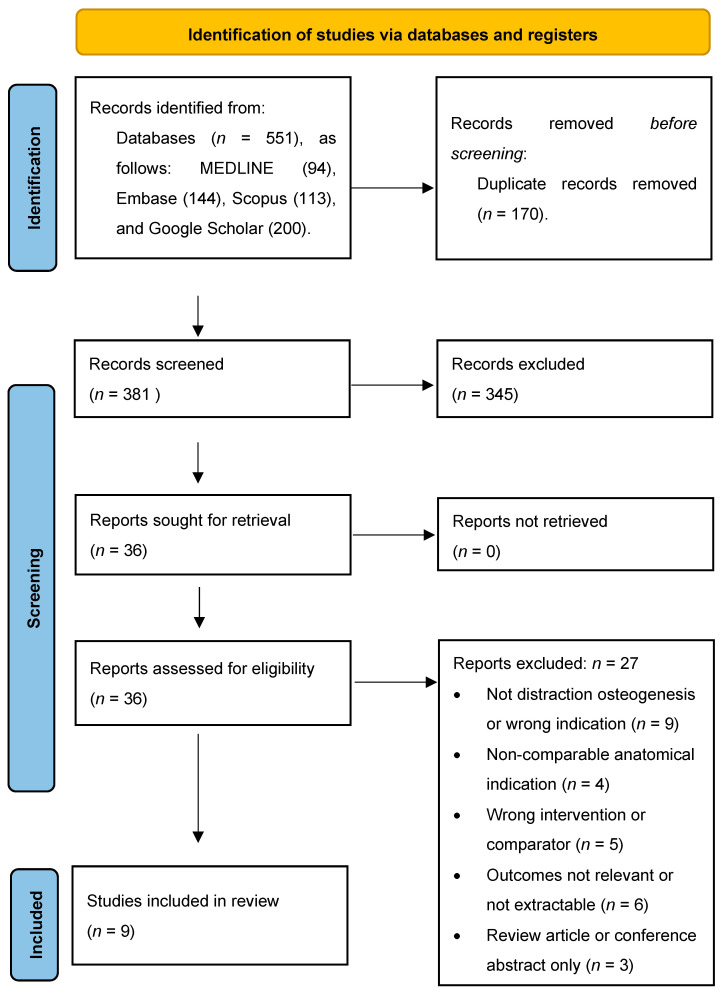
PRISMA 2020 flow diagram illustrating the study selection process, including the number of records identified through database searching, screened, assessed for eligibility, and ultimately included in the systematic review.

A total of 551 records were identified through database searching, including MEDLINE (*n* = 94), Embase (*n* = 144), Scopus (*n* = 113), and Google Scholar (*n* = 200). Following the removal of 170 duplicate records, 381 records were screened based on title and abstract. Of these, 345 records were excluded, and 36 reports were sought for retrieval, all of which were successfully retrieved (*n* = 0 not retrieved). After full-text assessment, 27 reports were excluded due to the following reasons: not distraction osteogenesis or wrong indication (*n* = 9), non-comparable anatomical indication (*n* = 4), wrong intervention or comparator (*n* = 5), outcomes not relevant or not extractable (*n* = 6), and review article or conference abstract only (*n* = 3). Ultimately, 9 studies were included in the systematic review. The full study selection process is illustrated in Figure 1.

### 3.2. Overview of Included Studies

Nine studies involving 304 participants met the inclusion criteria. Four studies evaluated low-intensity pulsed ultrasound (LIPUS) as an adjunct in tibial distraction osteogenesis: Dudda et al. (2011) [20], Salem et al. (2014) [23], Simpson et al. (2017) [24] and Song et al. (2019) [25]. Together, these trials included 149 patients, of whom 75 received LIPUS and 74 underwent standard distraction or sham ultrasound.

Five studies examined biologic augmentation in femoral or tibial distraction osteogenesis or aseptic tibial non-union using platelet-rich plasma (PRP) and/or bone marrow-derived products: Latalski et al. (2011) [26], Kitoh et al. (2007) [27,28] in two companion reports, Lee et al. (2014) [29] and Rollo et al. (2020) [30]. Across these studies, 155 patients were included, with 72 receiving a biologic adjunct and 83 serving as comparators.

Overall, four studies were randomized controlled trials (Dudda et al. [20], Salem et al. [23], Simpson et al. [24], Lee et al. [29]) and five were retrospective cohorts (Song et al. [25], Latalski et al. [26], Kitoh et al. [27,28] and Rollo et al. [30]). Study-level characteristics are summarized in Table 1.

### 3.3. Risk of Bias Assessment

**Figure 2:**
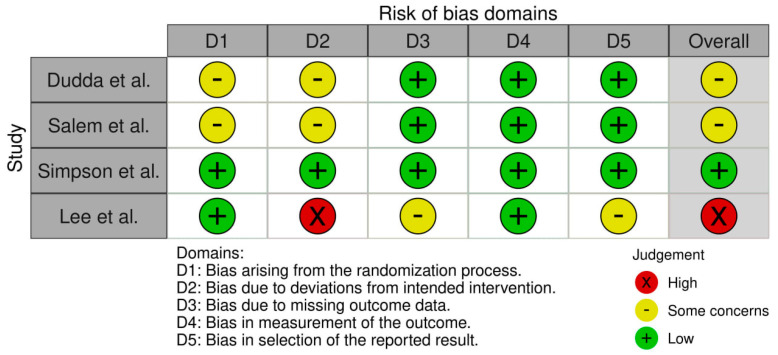
Risk of bias assessment for the included randomized controlled trials using the Cochrane Risk of Bias 2 (RoB 2) tool. The figure displays domain-specific judgments and the overall risk of bias for each randomized trial included in the review [20,23,24,29].

**Figure 3:**
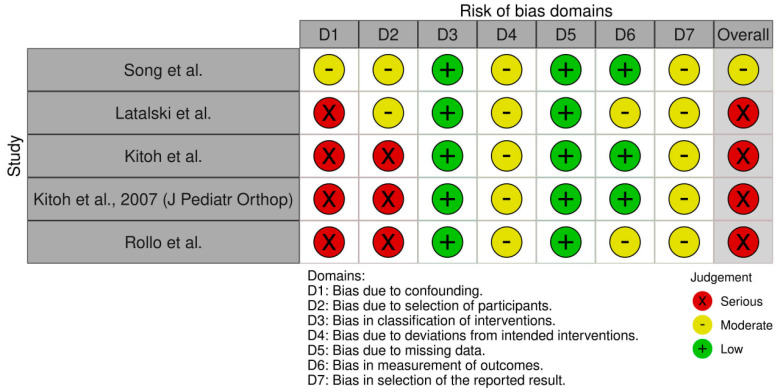
Risk of bias assessment for the included non-randomized cohort studies using the Risk Of Bias In Non-randomized Studies of Interventions (ROBINS-I) tool. The figure displays domain-specific judgments and the overall risk of bias for each non-randomized study included in the review [25,26,27,28,30].

The risk of bias assessment is summarized in Figure 2 and Figure 3.

Among the four randomized controlled trials (RCTs), one study was judged as having an overall low risk of bias, two studies were rated as having some concerns, and one study was classified as high risk of bias. The primary limitations identified among RCTs were related to insufficient reporting of the randomization process and lack of blinding, which contributed to concerns regarding deviations from intended interventions (Figure 2).

All included non-randomized studies were judged to have either moderate or serious risk of bias according to the ROBINS-I tool. The most prominent sources of bias were confounding and selection of participants, reflecting the inherent limitations of retrospective cohort designs (Figure 3).

Across studies, bias due to missing outcome data was generally low, as most studies reported complete or near-complete follow-up. Measurement of outcomes was also considered low risk in most cases, given the use of objective radiographic parameters such as healing index and external fixation index, although assessor blinding was inconsistently reported. Bias in the selection of reported results was commonly rated as moderate or some concerns, primarily due to the absence of pre-registered protocols or trial registration.

Overall, the methodological quality of the included evidence is limited by the predominance of non-randomized studies and methodological concerns identified in several randomized trials.

### 3.4. Participant Demographics

LIPUS trials enrolled predominantly young to middle-aged adults undergoing tibial lengthening. Mean ages ranged from 22.1 years in Song et al. [25] to 37.8 years in Simpson et al. [24], with male predominance in several series.

Biologic long-bone studies largely involved adolescents and young adults undergoing limb-lengthening or deformity correction. Latalski et al. [26] reported a mean age of 15.1 years; both Kitoh et al. [27,28] series had mean ages around 15–16 years; Lee et al. [29] studied patients with a mean age of 20 years. Rollo et al. [30] reported older adults (mean 42.9 years) with aseptic tibial non-union. Baseline demographic characteristics of all included studies are presented in Table 2.

### 3.5. Distraction and Adjunct Protocols

Where reported, long-bone distraction followed standard Ilizarov principles with distraction rates of approximately 1 mm/day in most tibial and femoral series (Dudda et al. [20], Salem et al. [23], Simpson et al. [24], Latalski et al. [26], both Kitoh et al. reports [27,28], Rollo et al. [30]). Lee et al. [29] used 0.75 mm/day for tibial lengthening over a nail.

Tibial distraction lengths in LIPUS trials ranged from 44 ± 23 mm (Simpson et al. [24]) to 82 mm (70–105 mm) in Song et al. [25] Biologic long-bone studies reported similar or slightly greater lengths (e.g., 5.55 ± 1.47 cm in Latalski et al. [26]; 83 ± 12.3 mm in short-stature patients and 37 ± 4.9 mm in limb-length discrepancy in the Bone report by Kitoh et al. [27]; 89.5 ± 13.6 mm in the JPO cohort [28]; 58 mm in Lee et al. [29]). Rollo et al. [30] treated shorter defects of 25 ± 7.44 mm in tibial non-union but with much longer overall healing times. Intervention and distraction protocol characteristics across all included studies are summarized in Table 3.

### 3.6. Healing Outcomes

#### 3.6.1. Healing Index

Healing index (HI), reported in days/cm or convertible months/cm, was the most consistent consolidation metric in long-bone distraction. with detailed values summarized in Table 4.

In LIPUS trials, Dudda et al. [20] reported an HI of 32.8 ± 13.1 days/cm (≈1.1 ± 0.44 months/cm), and Salem et al. [23] reported a similar value of 33 days/cm (SD not reported). In contrast, Simpson et al. [24] reported a regenerative maturation index of 65.8 ± 24.7 days/cm in a multicentre RCT. Song et al. analyzed consolidation by cortices in tibial lengthening over the nail, reporting separate healing indices for the anterior (36.6 days/cm), posterior (24.3 days/cm), medial (32.5 days/cm), and lateral (29.4 days/cm) cortices, along with an external fixation index of 29.4 days/cm. Statistically significant reductions in healing index were observed in the anterior (*p* < 0.001) and medial (*p* = 0.002) cortices compared with controls. The study did not report a single composite healing index, which precludes direct numerical comparison with studies reporting unified mean values.

Biologic long-bone studies showed a tighter cluster of healing indices. Latalski et al. [26] reported 29.9 ± 6.81 days/cm in PRP-treated femoral and tibial segments. The Bone article by Kitoh et al. [27] described HIs of 28.6 ± 7.07 days/cm in short-stature patients and 34.0 ± 4.18 days/cm in limb-length discrepancy. The JPO report in achondroplasia/hypochondroplasia found an HI of 27.1 ± 6.89 days/cm. Lee et al. [29] reported cortical healing indices of 0.81–1.14 months/cm and a weightbearing index of 0.89 months/cm, corresponding roughly to mid-20 s to low-30 s days/cm, though no single mean ± SD in days/cm was provided. Rollo et al. [30] discussed an external fixation index within ASAMI criteria but did not state a classic HI in days/cm.

Overall, LIPUS tibial trials show divergent HIs (two series around 33 days/cm vs. one multicentre RCT at ~66 days/cm), whereas biologic long-bone series consistently report HIs between ~27 and 34 days/cm across different bones and indications.

#### 3.6.2. Total Healing Time

Time to radiographic healing or frame removal mirrored the underlying indication. In tibial LIPUS series, mean healing times ranged from 165 days (Dudda et al. [20]) to 260–257 days (Salem et al. [23], Simpson et al. [24]). Latalski et al. [26] reported a mean healing time of 189.5 days in adolescents receiving PRP during femoral and tibial lengthening. Rollo et al. [30], working in aseptic tibial non-union, observed much longer healing durations of 456.6 ± 220 days, despite adjunctive PRP or HBO, reflecting the greater biological challenge of non-union compared with planned lengthening.

### 3.7. Complications, Bone Density and Functional Outcomes

Complications were reported inconsistently but were in keeping with expected external-fixation morbidity rather than clearly linked to LIPUS or biologic adjuncts. Dudda et al. [20] noted postoperative complications in two patients (arm not clearly specified). Latalski et al. [26] reported two minor infections in PRP-treated limbs. Lee et al. [29] described superficial infections in nine of 20 patients, without clear separation by adjunct status. Both Kitoh et al. reports documented isolated complications in single patients [27,28]. Rollo et al. [30] reported wire loosening, skin inflammation, skin retraction and delayed consolidation as typical Ilizarov-related issues. Quantitative bone density data were sparse. Salem et al. [23] reported a mean increase in bone density of 0.28 ± 0.12 units in LIPUS-treated tibial segments. Song et al. [25] provided cortical consolidation ranges but not a global density metric. Biologic long-bone studies relied on serial radiographs (and in some cases Doppler ultrasound) to judge regeneration quality without standardized numeric density reporting.

Functional outcomes and pain were best documented in ankle distraction and non-union series. Rollo et al. [30] used ASAMI criteria and SF-12 scores to evaluate tibial non-union outcomes. Numerical details were incompletely extractable; in this dataset they are therefore reported qualitatively (improvement or stability) rather than recalculated. Complications and secondary outcomes across all included studies are summarized in Table 5.

## 4. Discussion

### 4.1. Principal Findings

This systematic review evaluated nine clinical studies investigating biophysical and biologic adjuncts in distraction osteogenesis. Overall, the available evidence remains heterogeneous and methodologically limited. While several studies reported reductions in healing index or consolidation time, findings were inconsistent across anatomical sites, fixation strategies, and adjunct types. Randomized controlled trials assessing LIPUS demonstrated variable or negligible benefit, whereas biologic interventions such as PRP, BMAC, culture-expanded mesenchymal stem cells, and HBO showed promising regenerative signals but were largely supported by small trials or observational cohorts. Consequently, the certainty of evidence remains constrained, and definitive conclusions regarding superiority of one adjunct class over another cannot be drawn.

### 4.2. Comparative Interpretation: LIPUS Versus Biologic Adjuncts

The biological plausibility of LIPUS in distraction osteogenesis is compelling. As a mechanotransduction-based therapy, LIPUS theoretically enhances signaling pathways involved in regeneration formation. However, clinical data have not consistently demonstrated meaningful acceleration of consolidation. A meta-analysis of randomized trials in distraction osteogenesis reported no statistically significant reduction in treatment time or complication rate [31], and larger fracture-healing trials such as the TRUST study failed to show benefit in radiographic or functional outcomes [32]. Subsequent high-quality systematic reviews similarly concluded that LIPUS does not meaningfully reduce time to union or improve patient-important outcomes [26]. These findings mirror the variability observed within the distraction osteogenesis literature.

In contrast, biologic strategies aim to directly augment the cellular and molecular environment of the distraction gap. PRP delivers concentrated growth factors, BMAC and cell-based therapies provide osteogenic potential, and HBO aims to enhance the regenerative environment. Some individual trials have demonstrated improved consolidation parameters or earlier weight-bearing with biologic augmentation [29]. Nevertheless, these studies are often underpowered and methodologically heterogeneous. The broader orthopedic literature reveals similarly inconsistent outcomes for PRP and cell-based therapies, largely attributable to variability in preparation methods, cell concentrations, activation protocols, and delivery techniques [33,34]. Recent meta-analyses evaluating stem cell therapies in fracture healing have failed to demonstrate consistent improvements in union rates or pain reduction [35], underscoring the translational gap between promising preclinical data and clinical effectiveness.

Collectively, biologic adjuncts may offer more direct regenerative augmentation compared with LIPUS, which relies on amplification of endogenous mechanical signaling. However, current clinical evidence does not allow definitive determination of superiority.

### 4.3. Mechanistic Considerations

Distraction osteogenesis is inherently a mechanotransduction-driven process. Gradual tensile strain activates a coordinated cascade of osteogenic and angiogenic signaling, with tightly coupled vascular invasion and mineralization at the regenerate front [31,36,37]. Because distraction osteogenesis already incorporates substantial endogenous mechanical stimulation as a core element of its biological mechanism, it is plausible that additional mechanical amplification via LIPUS may offer diminishing incremental benefit, although this hypothesis requires direct clinical validation. In contrast, exogenous cell or growth factor supplementation may theoretically provide additive osteogenic stimuli through pathways distinct from mechanotransduction; however, the magnitude and clinical relevance of this additive effect remain to be established. However, successful regeneration formation depends on coordinated interactions among mechanical strain, vascular ingrowth, and endogenous progenitor activation rather than any single supplemented factor [38]. The complexity of this biological interplay may partially explain why adjunctive interventions demonstrate inconsistent clinical impact despite strong mechanistic rationale.

### 4.4. Variability Across Clinical Indications

The included studies encompassed a clinically diverse range of indications, including limb lengthening for short stature or limb-length discrepancy, deformity correction, congenital conditions such as achondroplasia and hypochondroplasia, and the management of aseptic tibial non-union. These indications differ meaningfully in their underlying biological context and healing dynamics, which warrants stratified interpretation of the findings. In planned lengthening procedures involving otherwise healthy bone, as represented by the LIPUS trials and the studies by Latalski et al. and Kitoh et al., the regenerate forms within a controlled mechanical environment with intact periosteal and endosteal contributions. Reported healing indices in these cohorts clustered within a comparable range of approximately 27 to 35 days per centimeter for biologic adjuncts, suggesting relatively consistent regenerative behavior under standardized distraction conditions. In contrast, achondroplasia and hypochondroplasia involve constitutionally altered chondro-osseous biology, which may influence both baseline regenerative behavior and responsiveness to biologic augmentation, as reflected in the Kitoh et al. JPO cohort. Aseptic tibial non-union, addressed in the Rollo et al. study, represents a fundamentally different biological scenario characterized by an already-compromised local healing environment, which is reflected in the substantially longer healing duration of 456.6 days observed in that cohort despite adjunctive therapy. These differences limit direct cross-indication comparison and underscore the need for future trials to stratify outcomes by clinical indication rather than pooling heterogeneous populations under a unified analytical framework.

### 4.5. Heterogeneity in Distraction Osteogenesis Research

A major barrier to evidence synthesis in this field is profound methodological heterogeneity. Studies vary substantially in anatomical site (long bone, alveolar, joint), fixation strategy (external fixation, lengthening over nail, plating), distraction protocol, timing of adjunct administration, and outcome reporting. Even the healing index—the most commonly used measure of consolidation efficiency—lacks a standardized definition. Some authors calculate it as total external fixation time per centimeter (external fixation index), whereas others define it as consolidation time per centimeter, leading to widely discrepant reported values [39]. A recent scoping review identified extensive variability in outcome definitions and measurement tools across lower-limb lengthening studies, with no consensus regarding radiographic, functional, or complication reporting [40]. Such heterogeneity precluded meaningful quantitative pooling in the present review and necessitated a structured narrative synthesis approach.

### 4.6. Methodological Quality and Risk of Bias

The evidence base for adjunctive interventions in orthopedic surgery is inherently constrained by methodological limitations. Many randomized trials are underpowered, lack adequate blinding due to the nature of surgical interventions, and demonstrate incomplete allocation concealment, all of which may inflate treatment effect estimates [41]. Publication bias further compounds these concerns, as positive findings are more likely to be published and cited [42]. Observational cohort studies—representing a substantial portion of the distraction osteogenesis literature—are additionally susceptible to selection and confounding biases that tend to overestimate treatment effects [43]. These methodological limitations align with the risk of bias assessment performed in the present review and temper confidence in reported effect sizes.

### 4.7. Clinical Implications

From a clinical standpoint, adjunctive therapies in distraction osteogenesis should be considered cautiously. While biologic and biophysical interventions may offer potential benefits in selected high-risk cases—such as delayed consolidation or compromised vascular environments—current evidence does not support routine adoption as standard of care. Moreover, the economic and logistical implications of daily LIPUS use or cell-based interventions must be weighed against the uncertain magnitude of clinical benefit. Individualized decision making based on patient risk profile, anatomical site, and resource availability remains prudent.

### 4.8. Strengths and Limitations of the Review

This review was prospectively registered, conducted in accordance with PRISMA 2020 guidelines, and included a structured risk of bias assessment. By comparatively evaluating biophysical and biologic adjunct classes within a single synthesis, it provides a comprehensive overview of available clinical evidence.

However, several limitations must be acknowledged. The small number of randomized trials, predominance of observational designs, heterogeneity in outcome definitions, and restriction to English-language publications limit the strength of conclusions. The absence of quantitative meta-analysis, while methodologically justified due to heterogeneity, may limit precision in effect estimation.

### 4.9. Future Research Directions

Future investigations in distraction osteogenesis should prioritize adequately powered randomized controlled trials with standardized definitions of healing index and consolidation time. Trials should incorporate patient-centered outcomes, including quality of life and functional recovery, which remain underreported in the current literature. Head-to-head comparisons between biophysical and biologic adjuncts, alongside cost-effectiveness analyses, would further clarify their relative value. Improved adherence to CONSORT reporting standards will be essential to enhance transparency and reproducibility [44].

## 5. Conclusions

Although biologic and biophysical adjuncts in distraction osteogenesis demonstrate compelling biological rationale, current clinical evidence remains heterogeneous and methodologically limited. While biologic strategies may offer theoretical advantages through direct cellular and growth factor supplementation, and LIPUS provides a non-invasive mechanotransductive stimulus, neither approach can currently be endorsed as a universally effective means of reducing consolidation time. High-quality, standardized, and adequately powered trials are required to define their true clinical role.

## Figures and Tables

**Table 1:** Study-level characteristics.

Study	Design	Population/Indication	Anatomical Site	Fixation Method	Adjunct Type	Adjunct *n*/Total *n*	Comparator
Dudda et al., 2011 [20]	RCT	Tibial distraction osteogenesis	Right/left tibia	Regazzoni (10), Ilizarov (3), hybrid (3)	LIPUS (1.5 MHz)	16/36	Standard DO without LIPUS
Salem et al., 2014 [23]	RCT	Tibial distraction osteogenesis	Tibia	Ilizarov ring fixators	LIPUS (1.5 MHz)	12/21	Standard DO without LIPUS
Simpson et al., 2017 [24]	RCT	Tibial distraction osteogenesis	Tibia	Ilizarov ring fixators	LIPUS (1.5 MHz)	32/62	Sham/standard DO (no LIPUS)
Song et al., 2019 [25]	Retrospective cohort	Tibial DO, lengthening over nail	Tibia	Ilizarov frame + lengthening over nail	LIPUS (1.5 MHz)	15/30	Standard lengthening over nail without LIPUS
Lee et al., 2014 [29]	RCT	Tibial distraction osteogenesis	Tibia	Lengthening over nail with external fixator	BMAC + PRP	10/20	No-injection group (standard DO)
Latalski et al., 2011 [26]	Retrospective cohort	Femoral/tibial distraction osteogenesis	Femur and tibia	Unilateral and ring external fixators	PRP	9/19	Standard lengthening without PRP
Kitoh et al., 2007 (Bone) [27]	Retrospective cohort	Long-bone DO in short stature/LLD	Femur (14), tibia (18)	Monolateral external fixation	Culture-expanded BM cells + PRP	17/46	Standard DO without cell/PRP transplant
Kitoh et al., 2007 (JPO) [28]	Retrospective cohort	Achondroplasia/hypochondroplasia lower limb DO	Femur and tibia	Monolateral external fixation	Culture-expanded BM cells + PRP	11/20	Standard DO without cell/PRP transplant
Rollo et al., 2020 [30]	Retrospective cohort	Aseptic tibial non-union treated with Ilizarov	Tibia	Ilizarov technique	PRP or hyperbaric oxygen (HBO)	25/50	Standard Ilizarov protocol (no adjunct)

**Table 2:** Baseline demographic characteristics.

Study	Age (Mean ± SD or Range)	Sex M/F (*n*)	BMI (Mean ± SD)	Follow-Up Duration
Dudda et al., 2011 [20]	34.9 ± 14.7 (17–64)	14/2	NR	NR
Salem et al., 2014 [23]	32 (SD NR)	12/0	weight 77 kg; height 179 cm	NR
Simpson et al., 2017 [24]	37.8 ± 12.9	10/22	27.0 ± 4.7	9–15 months
Song et al., 2019 [25]	22.1 (17.5–34)	12/3	21.7 kg/m^2^	5.3 years (1.7–8.2)
Lee et al., 2014 [29]	20 (16–28)	8/2	22 (18–26)	24–34 months
Latalski et al., 2011 [26]	15.1 ± 1.96 (12–18)	4/5	NR	NR
Kitoh et al., 2007 (Bone) [27]	15.8 (SD NR)	10/7	NR	NR
Kitoh et al., 2007 (JPO) [28]	16.3 ± 3.09	7/4	NR	NR
Rollo et al., 2020 [30]	42.86 ± 6.23 (16–72)	18/7	NR	NR

NR: not reported.

**Table 3:** Intervention and distraction protocol characteristics.

Study	Adjunct	Ultrasound Frequency/Biologic Type	Distraction Rate	Distraction Length (Mean ± SD)	Treatment Duration (Where Given)
Dudda et al., 2011 [20]	LIPUS	1.5 MHz	NR	69.9 mm	165.1 ± 95.7 days (total)
Salem et al., 2014 [23]	LIPUS	1.5 MHz	NR	79 mm	260.7 days
Simpson et al., 2017 [24]	LIPUS	1.5 MHz	NR	44 ± 23 mm	256.6 ± 101.2 days
Song et al., 2019 [25]	LIPUS	1.5 MHz	NR	82 (70–105) mm	External fixation index 29.4 days/cm (16.5–44.8)
Lee et al., 2014 [29]	BMAC + PRP	BMAC + PRP injections	0.75 mm/day	58 mm	NR
Latalski et al., 2011 [26]	PRP	PRP injection	1 mm/day	5.55 ± 1.47 cm	189.5 days
Kitoh et al., 2007 (Bone) [27]	BM cells + PRP	Culture-expanded BM cells + PRP	1 mm/day	83 ± 12.3 mm (short stature); 37 ± 4.9 mm (LLD)	NR
Kitoh et al., 2007 (JPO) [28]	BM cells + PRP	Culture-expanded BM cells + PRP	1 mm/day	89.5 ± 13.6 mm	NR
Rollo et al., 2020 [30]	PRP/HBO	PRP or HBO as callus accelerator	1 mm/day	25 ± 7.44 mm	456.6 ± 220 days (healing time)

NR: not reported.

**Table 4:** Healing indices and healing times.

Study	Context	Reported Healing Index	Healing Time/Treatment Time (Mean ± SD)
Dudda et al., 2011 [20]	Tibial DO + LIPUS	32.8 ± 13.1 days/cm (≈1.1 ± 0.44 months/cm)	165.1 ± 95.7 days (45–373 days)
Salem et al., 2014 [23]	Tibial DO + LIPUS	33 days/cm (SD NR)	260.7 days (SD NR)
Simpson et al., 2017 [24]	Tibial DO + LIPUS	65.8 ± 24.7 days/cm	256.6 ± 101.2 days
Song et al., 2019 [25]	Tibial lengthening over nail + LIPUS	Cortical healing indices (days/cm): anterior 36.6 (21.7–51.6), posterior 24.3 (15.3–43.0), medial 32.5 (19.5–47.6), lateral 29.4 (16.5–44.8)	External fixation index 29.4 days/cm (16.5–44.8)
Latalski et al., 2011 [26]	Femur/tibia DO + PRP	29.9 ± 6.81 days/cm	189.5 days (SD NR)
Kitoh et al., 2007 (Bone) [27]	Long-bone DO + BM cells + PRP	28.6 ± 7.07 days/cm (short stature); 34.0 ± 4.18 days/cm (LLD)	NR
Kitoh et al., 2007 (JPO) [28]	Lower limb DO + BM cells + PRP	27.1 ± 6.89 days/cm	NR
Lee et al., 2014 [29]	Tibial DO + BMAC + PRP	Cortical HI: 0.81–1.14 months/cm; weightbearing index 0.89 months/cm	NR
Rollo et al., 2020 [30]	Tibial non-union + PRP/HBO	External fixation index conceptually reported; no explicit days/cm	456.6 ± 220 days

Values are reported as in the original studies. SD, standard deviation; NR, not reported.

**Table 5:** Complications and secondary outcomes.

Study	Complications	Bone Density Numeric	Functional Scores Used
Dudda et al., 2011 [20]	2 postoperative complications (arm unclear)	NR	None specified
Salem et al., 2014 [23]	Not clearly separated by arm	Δ density 0.28 ± 0.12	None specified
Simpson et al., 2017 [24]	Not clearly reported per arm	NR	None specified
Song et al., 2019 [25]	Count of complications reported (no per-arm breakdown)	Cortical ranges only	None specified
Lee et al., 2014 [29]	Superficial infections in 9/20 (arm unclear)	NR	None specified
Latalski et al., 2011 [26]	2 minor infections in PRP-treated limbs	NR	None specified
Kitoh et al., 2007 (Bone) [27]	2 patients with complications (arm unclear)	NR	None specified
Kitoh et al., 2007 (JPO) [28]	1 patient with complications (arm unclear)	NR	None specified
Rollo et al., 2020 [30]	Wire loosening, skin inflammation, skin retraction, delayed consolidation (per-arm split unclear)	NR	SF-12, ASAMI (tools used, data not pooled)

NR: not reported.

## Data Availability

No new data were created or analyzed in this study.

## Supplementary Materials

The following supporting information can be downloaded at: https://www.mdpi.com/article/10.3390/jcm15124417/s1, Table S1: PRISMA 2020 Checklist [45].
