# Supplementary material for: Adjunctive Therapies in Long-Bone Distraction Osteogenesis: Clinical Evidence for Biophysical and Biologic Treatment Strategies
Source: J Clin Med. 2026 Jun 7;15(12):4417. doi: 10.3390/jcm15124417 (PMC13301968; doi:10.3390/jcm15124417)
Supplement: Supplementary file 1 [file jcm-15-04417-s001.zip › jcm-4298591-supplementary.pdf]

PRISMA 2020 Checklist

Section and Topic	Item #	Checklist item	Location where item is reported
**TITLE**			
Title	1	Identify the report as a systematic review.	Page 1
**ABSTRACT**			
Abstract	2	See the PRISMA 2020 for Abstracts checklist.	Page 1
**INTRODUCTION**			
Rationale	3	Describe the rationale for the review in the context of existing knowledge.	Page 1–2, Section 1 (Introduction), paragraphs 1–4.
Objectives	4	Provide an explicit statement of the objective(s) or question(s) the review addresses.	Page 2, Section 1 (Introduction), final paragraph
**METHODS**			
Eligibility criteria	5	Specify the inclusion and exclusion criteria for the review and how studies were grouped for the syntheses.	Page 2–3, Section 2.2 (Population), 2.3 (Interventions), 2.4 (Comparators), and 2.5 (Study Design)
Information sources	6	Specify all databases, registers, websites, organisations, reference lists and other sources searched or consulted to identify studies. Specify the date when each source was last searched or consulted.	Page 3, Section 2.6 (Information Sources and Search Strategy). MEDLINE, Embase, Scopus, and Google Scholar were searched from database inception to 6 October 2025.
Search strategy	7	Present the full search strategies for all databases, registers and websites, including any filters and limits used.	Page 3, Section 2.6 (Information Sources and Search Strategy), paragraph 2. Full Boolean search strings for each adjunct modality are presented.
Selection process	8	Specify the methods used to decide whether a study met the inclusion criteria of the review, including how many reviewers screened each record and each report retrieved, whether they worked independently, and if applicable, details of automation tools used in the process.	Page 3, Section 2.7 (Study Selection). Two reviewers independently screened titles, abstracts, and full texts; disagreements

Section and Topic	Item #	Checklist item	Location where item is reported
			resolved by consensus.
Data collection process	9	Specify the methods used to collect data from reports, including how many reviewers collected data from each report, whether they worked independently, any processes for obtaining or confirming data from study investigators, and if applicable, details of automation tools used in the process.	Page 3, Section 2.8 (Data Extraction). Two reviewers independently extracted data using a standardized form; disagreements resolved by consensus.
Data items	10a	List and define all outcomes for which data were sought. Specify whether all results that were compatible with each outcome domain in each study were sought (e.g. for all measures, time points, analyses), and if not, the methods used to decide which results to collect.	Page 3–4, Section 2.9 (Outcomes), including primary (healing index) and secondary outcomes (complications, radiological indicators, bone density, functional outcomes).
	10b	List and define all other variables for which data were sought (e.g. participant and intervention characteristics, funding sources). Describe any assumptions made about any missing or unclear information.	Page 3, Section 2.8 (Data Extraction). Variables included study design, demographics, indication, anatomical site, fixation method, adjunct protocol, distraction parameters, and outcome measures.
Study risk of bias assessment	11	Specify the methods used to assess risk of bias in the included studies, including details of the tool(s) used, how many reviewers assessed each study and whether they worked independently, and if applicable, details of automation tools used in the process.	Page 4, Section 2.10 (Risk of Bias Assessment). RoB 2 for randomized trials and ROBINS-I for non-randomized studies, applied independently by two reviewers.
Effect measures	12	Specify for each outcome the effect measure(s) (e.g. risk ratio, mean difference) used in the synthesis or presentation of results.	Page 3–4, Section 2.9 (Outcomes).

Section and Topic	Item #	Checklist item	Location where item is reported
			Healing index expressed as days per centimeter; consolidation time in days; complication rates; functional and radiographic outcomes.
Synthesis methods	13a	Describe the processes used to decide which studies were eligible for each synthesis (e.g. tabulating the study intervention characteristics and comparing against the planned groups for each synthesis (item #5)).	Page 4, Section 2.11 (Data Synthesis), paragraph 1.
	13b	Describe any methods required to prepare the data for presentation or synthesis, such as handling of missing summary statistics, or data conversions.	Page 4, Section 2.11 (Data Synthesis), paragraph 2. Heterogeneity in units and variance reporting precluded data conversion and pooled estimation.
	13c	Describe any methods used to tabulate or visually display results of individual studies and syntheses.	Page 4, Section 2.11 (Data Synthesis), final paragraph. Findings organized as structured narrative synthesis by adjunct type and outcome category, with summary tables (Tables 1–5).
	13d	Describe any methods used to synthesize results and provide a rationale for the choice(s). If meta-analysis was performed, describe the model(s), method(s) to identify the presence and extent of statistical heterogeneity, and software package(s) used.	Page 4, Section 2.11 (Data Synthesis). Quantitative meta-analysis was planned but not performed due to substantial clinical and methodological heterogeneity; narrative synthesis was conducted in accordance with PRISMA 2020

Section and Topic	Item #	Checklist item	Location where item is reported
			recommendations.
	13e	Describe any methods used to explore possible causes of heterogeneity among study results (e.g. subgroup analysis, meta-regression).	Not applicable — no quantitative synthesis was performed. Sources of heterogeneity are discussed narratively in Section 4.5.
	13f	Describe any sensitivity analyses conducted to assess robustness of the synthesized results.	Not applicable — no quantitative synthesis was performed.
Reporting bias assessment	14	Describe any methods used to assess risk of bias due to missing results in a synthesis (arising from reporting biases).	Not formally assessed due to the absence of quantitative synthesis; potential publication bias is discussed narratively in Section 4.6 (Methodological Quality and Risk of Bias).
Certainty assessment	15	Describe any methods used to assess certainty (or confidence) in the body of evidence for an outcome.	Page 4, Section 2.10 (Risk of Bias Assessment). Certainty of evidence was appraised qualitatively through study-level risk of bias using RoB 2 and ROBINS-I, with overall certainty discussed in Sections 4.1 and 4.6. Formal GRADE was not applied given the narrative synthesis design.
**RESULTS**			
Study selection	16a	Describe the results of the search and selection process, from the number of records identified in the search to the number of studies included in the review, ideally using a flow diagram.	Page 4–5, Section 3.1 (Study

PRISMA 2020 Checklist

Section and Topic	Item #	Checklist item	Location where item is reported
			Selection) and Figure 1 (PRISMA 2020 flow diagram).
	16b	Cite studies that might appear to meet the inclusion criteria, but which were excluded, and explain why they were excluded.	Page 5, Section 3.1 (Study Selection). Reasons for exclusion of 27 reports at full-text review are listed within the flow diagram.
Study characteristics	17	Cite each included study and present its characteristics.	Page 5, Section 3.2 (Overview of Included Studies) and Table 1 (Study-level characteristics).
Risk of bias in studies	18	Present assessments of risk of bias for each included study.	Page 5–6, Section 3.3 (Risk of Bias Assessment), Figures 2 and 3.
Results of individual studies	19	For all outcomes, present, for each study: (a) summary statistics for each group (where appropriate) and (b) an effect estimate and its precision (e.g. confidence/credible interval), ideally using structured tables or plots.	Page 6–7, Sections 3.4 (Participant Demographics), 3.5 (Distraction and Adjunct Protocols), 3.6 (Healing Outcomes), and 3.7 (Complications, Bone Density and Functional Outcomes), with Tables 2–5.
Results of syntheses	20a	For each synthesis, briefly summarise the characteristics and risk of bias among contributing studies.	Page 6–7, Sections 3.5–3.7. Summarized per intervention category alongside Tables 1–5.
	20b	Present results of all statistical syntheses conducted. If meta-analysis was done, present for each the summary estimate and its precision (e.g. confidence/credible interval) and measures of statistical heterogeneity. If comparing groups, describe the direction of the effect.	Not applicable — no meta-analysis was performed. Narrative results are presented in Sections 3.4–3.7.

Section and Topic	Item #	Checklist item	Location where item is reported
	20c	Present results of all investigations of possible causes of heterogeneity among study results.	Page 7, Section 3.6 (Healing Outcomes) and Section 4.5 (Heterogeneity in Distraction Osteogenesis Research).
	20d	Present results of all sensitivity analyses conducted to assess the robustness of the synthesized results.	Not applicable — no quantitative synthesis was performed.
Reporting biases	21	Present assessments of risk of bias due to missing results (arising from reporting biases) for each synthesis assessed.	Discussed narratively in Section 4.6 (Methodological Quality and Risk of Bias).
Certainty of evidence	22	Present assessments of certainty (or confidence) in the body of evidence for each outcome assessed.	Page 7, Section 4.1 (Principal Findings) and Section 4.6 (Methodological Quality and Risk of Bias).
**DISCUSSION**			
Discussion	23a	Provide a general interpretation of the results in the context of other evidence.	Page 7, Sections 4.1 (Principal Findings) and 4.2 (Comparative Interpretation: LIPUS Versus Biologic Adjuncts).
	23b	Discuss any limitations of the evidence included in the review.	Page 7–8, Sections 4.5 (Heterogeneity in Distraction Osteogenesis Research) and 4.6 (Methodological Quality and Risk of Bias).
	23c	Discuss any limitations of the review processes used.	Page 8, Section 4.8 (Strengths and Limitations of the Review).
	23d	Discuss implications of the results for practice, policy, and future research.	Page 8, Sections

Section and Topic	Item #	Checklist item	Location where item is reported
			4.7 (Clinical Implications) and 4.9 (Future Research Directions).
**OTHER INFORMATION**			
Registration and protocol	24a	Provide registration information for the review, including register name and registration number, or state that the review was not registered.	Page 2, Section 2.1 (Protocol and Registration). Prospectively registered with PROSPERO, registration number CRD420251125456.
	24b	Indicate where the review protocol can be accessed, or state that a protocol was not prepared.	Page 2, Section 2.1 (Protocol and Registration). Review protocol is accessible through the PROSPERO database under registration number CRD420251125456.
	24c	Describe and explain any amendments to information provided at registration or in the protocol.	No amendments were made to the registered protocol.
Support	25	Describe sources of financial or non-financial support for the review, and the role of the funders or sponsors in the review.	Page 8, Funding statement. This research received no external funding.
Competing interests	26	Declare any competing interests of review authors.	Page 8, Conflicts of Interest statement. The authors declare no conflict of interest.
Availability of data, code and other materials	27	Report which of the following are publicly available and where they can be found: template data collection forms; data extracted from included studies; data used for all analyses; analytic code; any other materials used in the review.	age 8, Data Availability Statement. All data extracted from the included studies are presented within the manuscript and its tables. No additional materials, code, or datasets were

PRISMA 2020 Checklist

Section and Topic	Item #	Checklist item	Location where item is reported
			generated. Original data are available in the published source studies cited in the references.

From: Page MJ, McKenzie JE, Bossuyt PM, Boutron I, Hoffmann TC, Mulrow CD, et al. The PRISMA 2020 statement: an updated guideline for reporting systematic reviews. BMJ 2021;372:n71. doi: 10.1136/bmj.n71

For more information, visit: http://www.prisma-statement.org/
